# Biographical Feature: Diane E. Griffin and Ann Palmenberg—luminaries in RNA virology

**DOI:** 10.1128/jvi.01164-25

**Published:** 2025-08-05

**Authors:** Robert F. Kalejta, Paul D. Friesen, Andrew Pekosz, Stacey Schultz-Cherry

**Affiliations:** 1 Institute for Molecular Virology, McArdle Laboratory for Cancer Research, University of Wisconsin-Madison 70033, Madison, Wisconsin, USA; 2 Department of Biochemistry, University of Wisconsin-Madison 200878, Madison, Wisconsin, USA; 3 W. Harry Feinstone Department of Molecular Microbiology & Immunology, Johns Hopkins Bloomberg School of Public Health 25802 , Baltimore, Maryland, USA; 4 Department of Host–Microbe Interactions, St. Jude Children’s Research Hospital, Memphis, Tennessee, USA; The University of Arizona, Tucson, Arizona, USA

**Keywords:** memorium

## TEXT

**Figure:**
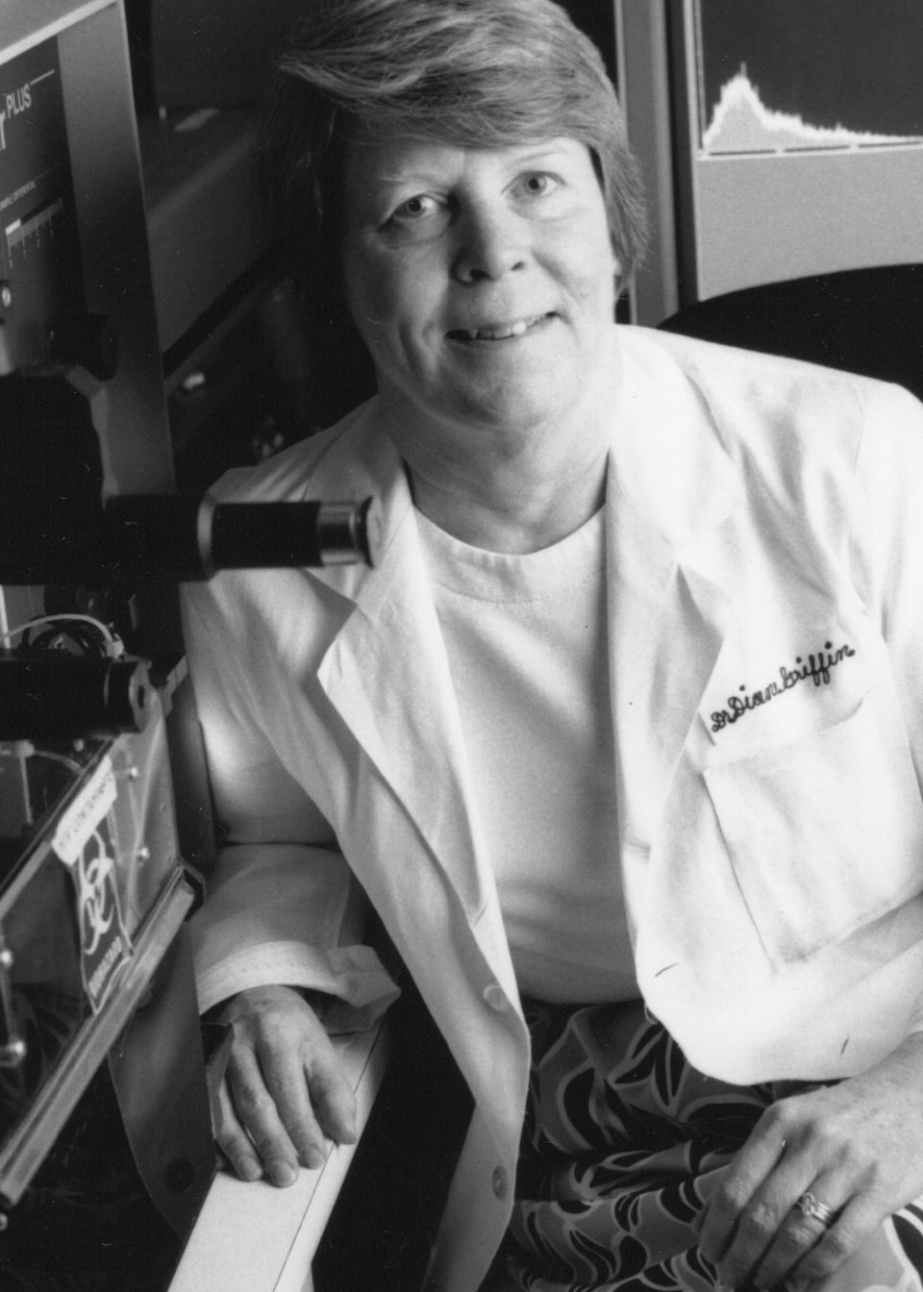


Over the past year, the virology community lost two luminaries in the field of RNA viruses: Diane Griffin (In Memoriam: Diane Edmund Griffin, MD, PhD, 1940-2024 | Johns Hopkins | Bloomberg School of Public Health) and Ann Palmenberg (Ann Palmenberg Obituary (1948 - 2025) - Madison / Nanuet, NY - Madison.com). Two incredible virologists, mentors, and people who truly made an impact on science and the community.

Dr. Diane Griffin, MD, PhD, was one of the most prominent scientific leaders of her generation. Her work was the first to demonstrate that measles virus infection causes death primarily by increasing susceptibility to other infections. She also showed that the measles virus leaves RNA particles after infection that may contribute to the lifelong protective immunity from measles. Diane also published seminal papers on how neurons were protected from death after virus infection and described non-lytic control of virus infection in neurons. During her decades at Johns Hopkins University, Diane was a leader in all that she did. She worked tirelessly for the research and public health communities leaving a tremendous legacy in the knowledge that she contributed to infectious diseases. Part of the legacy was the invaluable training she provided to the next generation of scientists and physicians, where she was known as an incomparable teacher, mentor, scientist, leader, and human being. Common themes when people talk about Diane are kindness, civility, and work ethics. Throughout her career, Diane achieved many accolades, including being an elected fellow of the American Association for the Advancement of Science as well as the Infectious Diseases Society of America. She was a frequent participant in the National Institutes of Health study sections, chairing the Special AIDS Study Section and co-chairing the Board of Scientific Counselors at the National Institute of Allergy and Infectious Diseases, and was Vice President of the US National Academy of Sciences. She edited the *Journal of Virology* from 1994 to 2004, was a past president of the American Society for Virology (ASV) and the American Society for Microbiology, and was inducted into the Maryland Women’s Hall of Fame in 2009. Read more about Dr. Griffin’s work.

**Figure:**
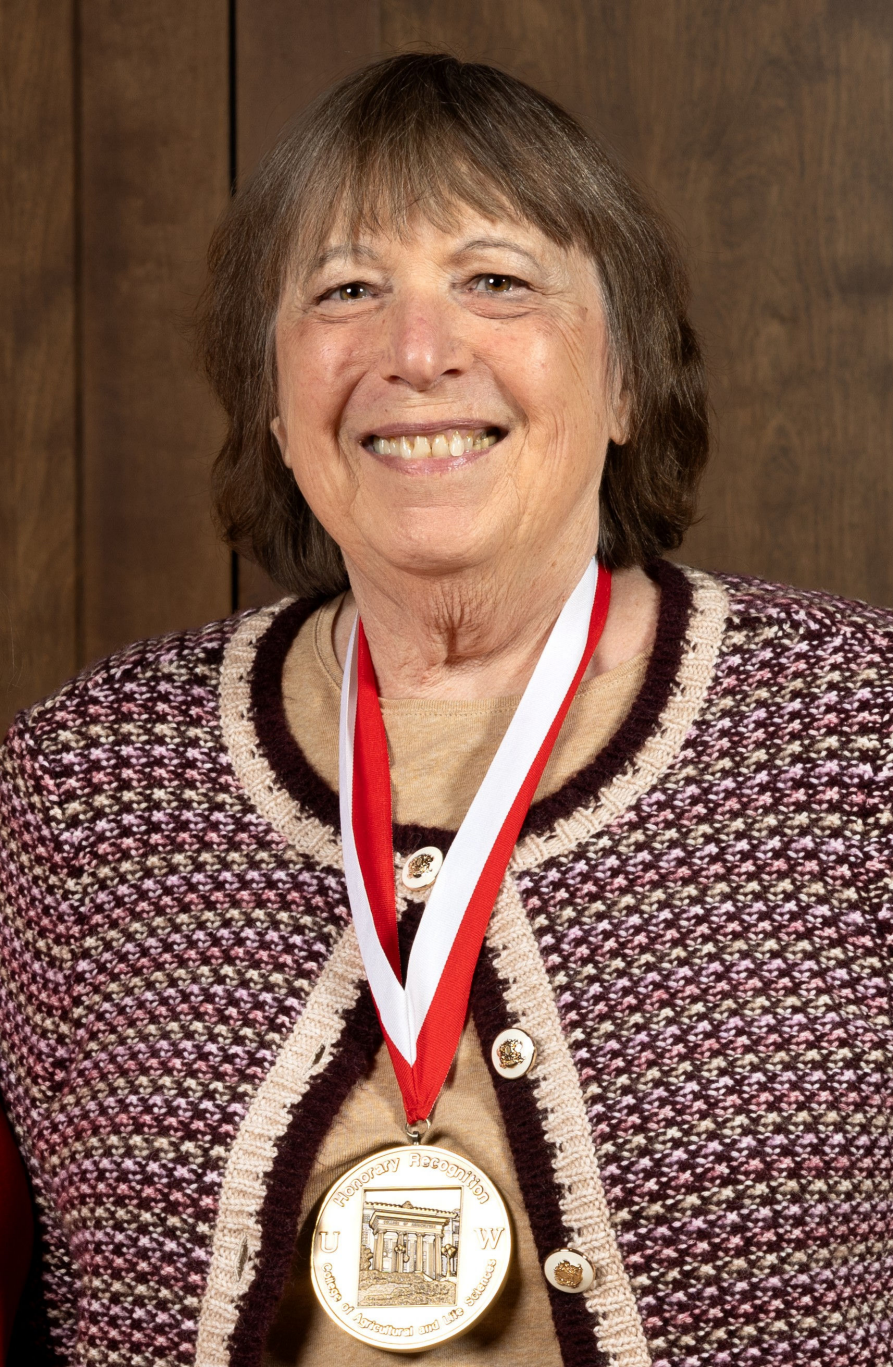


Dr. Ann Palmenberg, PhD, was a trailblazer in the field of RNA virology. Ann’s humble description of her work as simply “taking viruses apart and putting them back together” fails to capture her scientific brilliance, influence on the field, and unrivaled support of junior faculty and women in science. A renowned expert on the biochemistry of picornaviruses, her work solved the atomic structure of human rhinovirus C, which paved the way for new therapies and antivirals against virus-induced asthma. Ann was also the first to describe a way to make new types of live virus vaccines using viral internal ribosome entry sites (IRES). The discovery of the IRES still serves as the manufacturing basis for numerous biotechnological and pharmaceutical products and made for an interesting, personalized license plate for her automobile. During her decades at the University of Wisconsin-Madison (UW) as a Professor of Biochemistry, Ann received numerous awards from the university and the Wisconsin Alumni Research Foundation. She served as the Director of UW’s Institute for Molecular Virology for 15 years. She also enjoyed serving on the UW Athletic Board and was frequently found in the bleachers at Badger Big Ten football, basketball, and hockey games. She may be best recognized by the virology community for her dedication to the ASV, where she spent countless hours organizing memorable annual conferences. In 2007, Ann was elected ASV President and in recognition of her influential research and 30 years of devoted service to the Society, she received the 2024 Wolfgang & Patricia Joklik Distinguished Service Award from the ASV. Multiple awards were also established in her name during her lifetime, including the prestigious Ann Palmenberg Junior Investigator Award conferred annually by the ASV to junior virologists. Ann never did anything halfway. Her dedication to the virology community, her colleagues, her trainees, and her tremendous advocacy and support of junior faculty and women in science will be sorely missed. Read more about Dr. Palmenberg’s work.

